# User and Provider Experiences With Health Education Chatbots: Qualitative Systematic Review

**DOI:** 10.2196/60205

**Published:** 2025-06-13

**Authors:** Кyung-Eun (Anna) Choi, Sebastian Fitzek

**Affiliations:** 1 Health Services Research Group, Medical Images Analysis and Artificial Intelligence, Danube Private University, Steiner Landstraße 124, Krems an der Donau, 3500, Austria, 43 0734102289; 2 Center for Health Services Research, Brandenburg Medical University, Neuruppin, Germany; 3 Evidence-Based Practice in Brandenburg—A JBI Affiliated Group, Hochstraße 29Brandenburg an der Havel, 14770, Germany

**Keywords:** chatbot, health education, behavior change, user experience, privacy concerns, personalization, qualitative research.

## Abstract

**Background:**

Chatbots, as dialog-based platforms, have the potential to transform health education and behavior-change interventions. Despite the growing use of chatbots, qualitative insights into user and provider experiences remain underexplored, particularly with respect to experiences and perceptions, adoption factors, and the role of theoretical frameworks in design.

**Objective:**

This systematic review of qualitative evidence aims to address three key research questions (RQs): (RQ1) user and provider experiences; (RQ2) facilitators and barriers to adoption; and (RQ3) role of theoretical frameworks.

**Methods:**

We systematically searched PubMed, the Cochrane Library, and ScienceDirect from January 1, 2018, to October 1, 2023, for English- or German-language, peer-reviewed qualitative or mixed methods studies. Studies were included if they examined users’ or providers’ experiences with chatbots in health education or behavior-change contexts. Two reviewers independently screened titles, abstracts, and full texts (Cohen κ=0.82). We used the Joanna Briggs Institute Critical Appraisal Checklist for quality assessment and conducted a reflexive thematic analysis following Braun and Clarke’s framework.

**Results:**

Among the 1754 records identified, 27 studies from 10 countries met the inclusion criteria, encompassing 241 qualitative-only participants and 10,802 mixed method participants (657 contributing qualitative data). For RQ1, users emphasized empathy and emotional connection. For RQ2, accessibility and ease of use emerged as facilitators, whereas trust deficits, technical glitches, and cultural misalignment were key barriers. For RQ3, the integration of behavior-change theories emerged as underutilized despite their potential to increase motivation.

**Conclusions:**

Chatbots demonstrate strong potential for health education and behavior-change interventions but must address privacy and trust issues, embed robust theoretical underpinnings, and overcome adoption barriers to fully realize their impact. Future directions should include evaluations of cultural adaptability and rigorous ethical considerations in chatbot design.

## Introduction

Chatbots are software applications designed for 2-way dialogue that simulate human conversation through text or speech and are increasingly integrated into health care systems to deliver health education and behavior-change interventions [1,2]. These applications provide tailored guidance and real-time support by leveraging the widespread availability of smartphones and internet connectivity, thereby delivering standalone health interventions [3]. Users receive timely information, reminders, and motivational messages that may improve health outcomes [4,5].

Despite their growing use, previous systematic reviews and meta-analyses have focused predominantly on quantitative outcomes, such as symptom reduction and behavioral compliance [1-3,6], leaving significant gaps in our understanding of the qualitative dimensions of chatbot interactions [7]. These gaps, namely, the limited qualitative insights into user experiences, the underutilization of theoretical frameworks in chatbot design, and an insufficient understanding of adoption factors, form the foundation of this study and directly inform our 3 research questions (RQs).

Established theoretical frameworks, such as the health belief model (HBM), the technology acceptance model (TAM), and social cognitive theory, offer valuable constructs for understanding and motivating behavior change [8-11]; however, these models are rarely integrated into current chatbot designs. This underutilization may limit chatbots’ capacity to enhance user engagement and sustain behavior change.

This review aims to synthesize qualitative insights into the following:

1. What are the experiences and perceptions of users and providers regarding chatbots in health education and behavior change?
2. What are the key facilitators and barriers affecting the adoption of healthcare chatbots?
3. How do theoretical frameworks guide the design and implementation of these chatbots?

## Methods

### Overview

This systematic review aligns with the meta-aggregation principles outlined by Lockwood et al [12] (adapted for qualitative evidence synthesis), which we have applied to the domain of health education and behavior change interventions delivered via conversation-based digital tools, including chatbots. This approach was chosen to explore in-depth user and provider experiences through a specific focus on qualitative data, directly addressing the RQs on perceptions, theoretical frameworks, and adoption facilitators and barriers. We adhered to the guidelines of the Joanna Briggs Institute (JBI) [13] for critical appraisal and reported our methods in accordance with the PRISMA (Preferred Reporting Items for Systematic Reviews and Meta-Analyses) flowchart and standard systematic review protocols. The review was registered with the Open Science Framework to ensure methodological transparency.

### Search Strategy

We searched PubMed, the Cochrane Library, and ScienceDirect from January 1, 2018, to October 1, 2023, for English- or German-language, peer-reviewed qualitative or mixed methods studies. A medical librarian assisted in developing the search strategy to ensure comprehensive coverage. Our search syntax included terms such as “chatbot,” “conversational agent,” “digital health interventions,” “qualitative,” and “mixed methods.” These terms were applied to titles, abstracts, and keywords to identify relevant studies examining conversation-based digital tools for health education or behavior change. The detailed search syntax is presented in Multimedia Appendix 1.

### Study Selection

Two reviewers (SF and KEC) independently screened all titles and abstracts (Cohen κ=0.82), retrieving full texts when either deemed an article potentially relevant. Discrepancies were resolved by discussion, obviating the need for a third reviewer. Mixed methods studies were included if they provided relevant qualitative data, which were prioritized over quantitative findings during synthesis to ensure a comprehensive understanding of user and provider experiences. The final inclusion criteria limited articles to those presenting qualitative data on conversation-based digital tools in health education or behavior change, with participants or providers offering experiential insights. References were tracked and managed via EndNote (Clarivate) and manual cross-checking. An overview of the complete selection process is shown in Multimedia Appendix 2, along with the PRISMA flow diagram.

### Inclusion and Exclusion Criteria

Studies were selected on the basis of the detailed inclusion and exclusion criteria outlined in Table 1. In brief, we included qualitative or mixed methods studies published in English or German between 2018 and 2023 that investigated user or provider experiences with conversation-based digital tools designed for health education or behavior change. These tools encompass various designations, including “chatbots,” “conversational agents,” and “digital health assistants,” all of which rely fundamentally on dialog-based engagement. This inclusive approach enables a comprehensive examination of automated conversational systems within health intervention contexts. The time frame (2018‐2023) was selected to capture recent developments in chatbot technology, while the language restriction was based on the research team’s proficiency. Studies were excluded if they lacked conversational functionality, did not provide qualitative data, or focused on domains unrelated to health education and behavior change. These criteria were systematically applied to ensure consistent study selection and reduce potential bias.

**Table 1.** Inclusion and exclusion criteria.

Criterion	Inclusion	Exclusion
Study type	Primary qualitative studies, mixed methods studies with qualitative components, and peer-reviewed articles	Purely quantitative studies, reviews, editorials, opinion pieces, conference abstracts, and non–peer-reviewed articles
Population	Patients and health consumers, health care professionals, adults (aged 18 years and older), adolescents (aged 12‐19 years) with specific health conditions	Studies focused exclusively on children and studies without direct user and provider perspectives
Intervention	Chatbots and conversational agents for health education, chatbots for behavior change, artificial intelligence–driven health dialog systems	Mobile apps without conversational features, static health information systems, and noninteractive digital tools
Outcomes	User experiences, provider experiences, perceptions of chatbot use, qualitative feedback on usability, and implementation insights	Only quantitative outcomes, technical performance metrics, and cost-effectiveness analyses
Language	English and German	All other languages
Publication period	Published between 2018 and 2023	Studies published before 2018 and after 2023
Context	Health care settings, health education contexts, and behavior change interventions	Non–health care settings, commercial customer service, and general technology evaluation
Study design quality	Clear methodological description, appropriate data collection methods, and rigorous analysis procedures	Poor methodological quality, insufficient description of methods, and lack of ethical considerations

### Data Extraction and Quality Appraisal

A standardized form was used to extract data on study design, participant demographics, chatbot features, outcomes, and key qualitative findings, ensuring consistency across studies. Two reviewers initially extracted data from 5 articles to establish a consistent approach before dividing the remaining articles; ongoing discussions resolved any ambiguities. The JBI Critical Appraisal Checklist for Qualitative Research was used to assess the methodological rigor and risk of bias (Multimedia Appendix 3). Rather than excluding studies failing to meet certain criteria, our appraisal informed the weighting of findings during synthesis.

### Data Analysis

We conducted reflexive thematic analysis [14], beginning with immersive reading and coding of text fragments and progressing through 6 phases: (1) familiarization, (2) initial coding, (3) theme development, (4) review, (5) definition and naming, and (6) reporting. Subsequent iterative rounds of coding and discussion refined the analysis, and the themes were validated against the context of each article. To ensure rigor, investigator triangulation was used, with both reviewers achieving high interrater reliability (Cohen κ=0.82 for the screening process), along with regular analytic meetings and reflective journaling to mitigate researcher bias. The identified themes were organized in alignment with our 3 RQs, and representative participant quotes were integrated to illustrate key findings and enhance the credibility of the analysis.

## Results

### Overview of Included Studies

**Figure 1.**
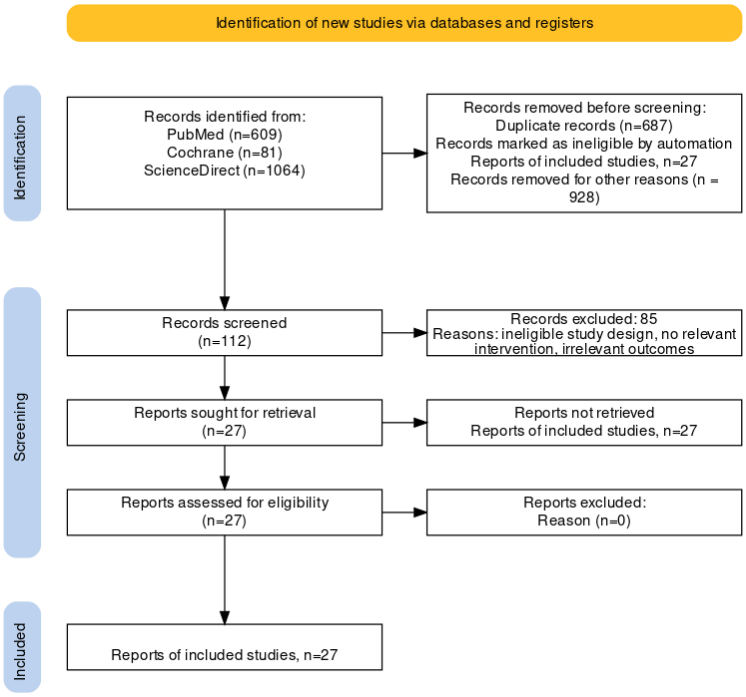
PRISMA (Preferred Reporting Items for Systematic Reviews and Meta-Analyses) flow diagram of the study selection process*.*

From the 1754 records identified, 27 articles from 10 countries satisfied our criteria, encompassing 169 participants in exclusively qualitative studies and 10,802 in mixed methods studies, of whom 657 contributed qualitative data (Figure 1). The studies varied in focus, encompassing chatbot design, technological innovations, behavior change, mental health, and user experience research. Participant ages ranged from adolescents to older adults, highlighting the broad adaptability of chatbots across diverse health contexts. The participants included patients, health care providers, and general users, with studies primarily using qualitative interviews or mixed methods designs. For a detailed summary of the study characteristics, see Table 2.

**Table 2.** Summary of study characteristics.

First author	Title	Year	Country	Aim of the study	Study design	Participants
Baptista et al [15]	Acceptability of an embodied conversational agent for type 2 diabetes self-management education and support	2020	Australia and New Zealand	To evaluate the acceptability of an embodied conversational agent, named Laura, used to deliver diabetes self-management education and support in the My Diabetes Coach (MDC) app.	Randomized controlled trial and mixed methods	93 participants in the intervention arm
Barnett et al [16]	Enacting “more-than-human” care: clients’ and counsellors’ views on the multiple affordances of chatbots in alcohol and other drug counseling	2021	Australia	The paper explores the experiences and perceptions of clients and counsellors of chatbots used in web-based alcohol and other drug counseling. It focuses on how these technologies afford (provide) or constrain online care and client interactions.	Mixed	20 clients (10 men and 10 women) and 8 counsellors (5 men and 3 women)
Beaudry et al [17]	Getting ready for adult healthcare: designing a chatbot to coach adolescents with special health needs through the transitions of care	2019	United States	To engage adolescents with chronic medical conditions using a chatbot and text messaging platform to promote skill attainment in self-care and ease the transition from pediatric to adult-focused care	Pilot study	13 adolescents with chronic conditions
Biro et al [18]	The effects of a health care chatbot’s complexity and persona on user trust, perceived usability, and effectiveness: mixed methods study	2023	Clemson University, Clemson, United States	To examine how various design elements impact the efficacy of a health care chatbot intended for educational purposes.	Mixed methods	71 university students aged 18‐26 years (28 males, 43 females; 69% Caucasian, 11.3% African American, 19.7% Asian)
Boggiss et al [19]	Improving the well-being of adolescents with type 1 diabetes during the COVID-19 pandemic: qualitative study exploring acceptability and clinical usability of a self-compassion chatbot	2023	Aotearoa, New Zealand	To assess the effectiveness and acceptance of a self-compassion chatbot (COMPASS) designed for adolescents with type 1 diabetes.	Qualitative	19 adolescents with type 1 diabetes and 11 diabetes health care professionals
Chang et al [20]	Why would you use medical chatbots? Interview and survey	2022	Taiwan	To understand the factors influencing individuals’ attitudes and intentions to use medical chatbots.	Two-stage mixed-method approach	20 interview participants and 205 survey respondents
Chen et al [5]	Developing a heart transplantation self-management support mobile health app in Taiwan: qualitative study	2020	Taiwan	To investigate the information needs of post–heart transplantation patients and develop a preliminary framework for a mobile health app to support their self-management.	Qualitative	17 post–heart transplantation patients and 4 health professionals
Galvão Gomes da Silva et al [21]	Experiences of a motivational interview delivered by a robot: qualitative study	2018	United Kingdom	To explore the experiences of individuals who have engaged in a motivational interview delivered by a social robot.	Qualitative	20 participants from the School of Psychology’s pool of research volunteers
Griffin et al [22]	A chatbot for hypertension self-management support: user-centered design, development, and usability testing	2023	United States	To incorporate users’ feedback into Medicagent through usability testing, leveraging the Information-Motivation-Behavioral skills model and the model of medication self-management	Mixed	10 adults with hypertension
Griffin et al [23]	Information needs and perceptions of chatbots for hypertension medication self-management: a mixed methods study	2021	Chapel Hill, North Carolina, United States	To understand information needs and perceptions toward using a chatbot to support hypertension medication self-management	Convergent mixed methods design	15 adults with hypertension
Han et al [24]	Preliminary evaluation of a conversational agent to support self-management of individuals living with posttraumatic stress disorder: interview study with clinical experts	2023	United States	To conduct a preliminary evaluation of the PTSDialogue, focusing on its usability and acceptance from the viewpoint of clinical experts.	Qualitative	10 clinical experts with experience in posttraumatic stress disorder care
Hurmuz et al [25]	Evaluation of a digital coaching system eHealth intervention: a mixed methods observational cohort study in the Netherlands	2022	The Netherlands	To assess the use, user experience, and potential health effects of a conversational agent-based eHealth platform among older adults.	Observational cohort study	51 older adults, 70.6% female, and average age of 65 years
Kornfield et al [26]	A text messaging intervention to support the mental health of young adults: user engagement and feedback from a field trial of an intervention prototype	2023	United States	To investigate young adults’ engagement and experiences with a prototype of an interactive text messaging program for managing mental health concerns.	User-centered design	48 individuals (66.7% female and 33.3% male)
Lin et al [27]	Exploring pictorial health education tools for long-term home care: a qualitative perspective	2020	Taiwan	To explore a theoretical framework for developing pictorial health education tools to enhance communication between medical and nonmedical staff in home care.	Grounded theory	6 designers, 5 medical staff (hospital director, supervisor, nurses), and 8 groups of home caregivers for intubated patients
Ly et al [28]	A fully automated conversational agent for promoting mental well-being: a pilot RCT using mixed methods	2017	Sweden	To map and synthesize qualitative evidence on the use of chatbots in health education and behavioral change settings.	Mixed methods	9 individuals, women (n=4) and men (n=5), mean age of 28.8 years
Mash et al [29]	Evaluating the implementation of the GREAT4 Diabetes WhatsApp chatbot to educate people with type 2 diabetes during the COVID-19 pandemic: convergent mixed methods study	2022	South Africa	To assess the implementation of the GREAT4Diabetes chatbot, focusing on adoption, appropriateness, acceptability, and other implementation outcomes.	Mixed methods	8158 people connected with the chatbot
Nadarzynski et al [30]	Acceptability of artificial intelligence (AI)-led chatbot services in healthcare: a mixed-methods study	2019	United Kingdom	To assess the acceptability of artificial intelligence–driven health chatbots and uncover challenges and driving factors impacting their use.	Mixed methods	29 university students for interviews and 215 individuals for survey
Papadopoulos et al [7]	Socially assistive robots in health and social care: acceptance and cultural factors. Results from an exploratory international internet-based survey	2022	United Kingdom	To explore registered nurses’ and midwives’ views on socially assistive robots and the impact of cultural dimensions on their acceptance.	Exploratory, cross-sectional, and descriptive study	1341 participants from 19 countries
Roman et al [31]	“Hey assistant, how can I become a donor?” The case of a conversational agent designed to engage people in blood donation	2020	Brazil	To develop and assess a conversational agent aimed at engaging people in blood donation.	User experience assessment study	50 participants (16 men and 34 women)
Schmidlen et al [32]	Patient assessment of chatbots for the scalable delivery of genetic counseling	2019	United States	To gather feedback on chatbots as new communication tools for facilitating genetic counseling.	Qualitative	62 participants in 6 focus groups
Scholten et al [33]	An empirical study of a pedagogical agent as an adjunct to an eHealth self-management intervention	2019	Netherlands	To investigate the support level technology, specifically a pedagogical agent, can provide to users of a self-guided positive psychology psycho-education.	Between-subjects experiment	230 psychology students
Siglen et al [34]	Ask Rosa – the making of a digital genetic conversation tool, a chatbot, about hereditary breast and ovarian cancer	2022	Norway	To design and develop a pilot version of the Rosa chatbot as a reliable source of information on hereditary breast and ovarian cancer.	Participatory methodology	58 participants including patient representatives, IT engineers, and medical staff
Svendsen et al [35]	One size does not fit all: participants’ experiences of the selfBACK app to support self management of low back pain	2022	Denmark and Norway	To investigate patients’ experiences with the selfBACK app for self-management of low back pain.	Qualitative interview study	26 participants from the selfBACK trial
Swendeman et al [36]	Feasibility and acceptability of mobile phone self-monitoring and automated feedback to enhance telephone coaching for people with risky substance use	2021	United States	To evaluate the feasibility and acceptability of mobile-phone delivered self-monitoring and feedback integrated into a health coaching intervention for risky drug use.	Mixed methods	20 participants with risky substance use
Ter Stal et al [37]	An embodied conversational agent in an eHealth self-management intervention for chronic obstructive pulmonary disease and chronic heart failure	2021	The Netherlands	To investigate users’ perceptions of an embodied conversational agent’s design in a real-life setting.	Mixed methods design	11 participants with chronic obstructive pulmonary disease and congestive heart failure
Escobar-Viera et al [38]	A chatbot-delivered intervention for optimizing social media use and reducing perceived isolation among rural-living LGBTQ+ youth	2023	United States	To evaluate REALbot, a chatbot designed to deliver an educational program to rural living LGBTQ+ youth to reduce perceived isolation.	Exploratory pilot study	20 adolescents aged 14‐20 years old, identifying as LGBTQ+
Wang et al [39]	Revealing the complexity of users’ intention to adopt healthcare chatbots: a mixed-method analysis of antecedent condition configurations	2023	China	To understand users’ attitudes and experiences with online health care chatbots.	Mixed methods	347 survey respondents and 12 participants in semistructured interviews

### RQ1: User and Provider Experiences

RQ1 explores the general experiences and perceptions of users and providers regarding chatbots in health education and behavior change, which is distinct from the specific adoption factors addressed in RQ2.

#### Empathy and Emotional Connection

The participants valued human-like interactions in chatbots, particularly for emotional support. Ly et al [28] noted that participants perceived chatbots as living characters capable of forming relationships, emphasizing their potential to mimic human guidance and empathy. However, providers expressed skepticism about chatbots’ empathetic capabilities. Nadarzynski et al [30] raised significant concerns, stating that chatbots are not capable of empathy, notably in recognizing users’ emotional states and tailoring responses accordingly, potentially compromising user engagement.

#### Trust and Privacy Concerns

Privacy and data security are significant concerns affecting trust in chatbots. A participant in the study by Nadarzynski et al [30] stated, “Some things are confidential and you wouldn’t just type it on the internet. You would want the confidentiality of [a doctor],” highlighting concerns over the chatbot’s ability to maintain the confidentiality expected in traditional medical interactions. These concerns highlight the tension between accessibility and data protection.

### RQ2: Facilitators and Barriers to Adoption

RQ2 investigates specific factors influencing the adoption of healthcare chatbots, distinguishing them from the broader experiences covered in RQ1.

#### Accessibility and Ease of Use

Round-the-clock availability and intuitive design were key facilitators. Boggiss et al [19] explicitly stated that chatbots offer unique advantages, including “24-hour availability, accessibility, remote delivery, scalability, and real-time personalized responses.”

#### Personalization

Personalization enhanced adoption. Chang et al [20] highlighted user desire for personalized health information, rather than generic content, to enhance relevance and usefulness. Boggiss et al [19] further supported this, noting that nearly all users wanted to customize chatbot interactions.

#### Trust Deficits

Trust issues, particularly those related to privacy, hinder adoption. Barnett et al [16] expressed concerns about confidentiality, noting uncertainty regarding who has access to chatbot interactions and thus preferring traditional doctor–patient confidentiality.

#### Technical Glitches

Technical issues such as app freezes and connectivity problems were barriers. Mash et al [29] explicitly described technical problems such as repeated messages and system overloads affecting message dissemination to users, significantly disrupting user experiences. Svendsen et al [35] reported that faulty synchronization and login issues impact chatbot use.

#### Cultural Misalignment

Chatbots’ inability to interpret cultural nuances posed obstacles. Papadopoulos et al [7] highlighted the chatbot’s difficulty in understanding cultural contexts, stating, “It may interfere with the patient, the patient may not be able to explain the problem to the robot because the robot does not know how people grow in cultures in places.”

### RQ3: Role of Theoretical Frameworks

RQ3 examines how theoretical frameworks of health behavior change and how technology acceptance guides chatbot design and implementation.

#### Underutilization of Behavior Change Models

While some studies have integrated frameworks such as the HBM, TAM, or social cognitive theory, many have not, missing opportunities to increase user motivation. Griffin et al [22] explicitly used the IDEAS (Integrate, Design, Assess, and Share) framework and information-motivation-behavioral skills model to inform chatbot design for hypertension management, demonstrating the positive impact of theory-driven approaches. Conversely, Nadarzynski et al [30] identified the absence of a clear theoretical foundation as problematic, noting risks such as incorrect self-diagnosis and inadequate care from theoretically ungrounded chatbots.

#### Integration With Theoretical Constructs

Studies incorporating theoretical constructs reported improved engagement. Griffin et al [22] positively illustrated the integration of behavioral theories, notably the IDEAS framework and the information-motivation-behavioral skills model, effectively informing users and motivating behavior change.

### Additional Analytical Themes

This section synthesizes insights from RQ1-RQ3 to illustrate how themes of empathy, trust, and theoretical grounding converge in the experiences of users and providers. Chatbots were seen as evolving interfaces capable of triaging minor questions before escalation, as highlighted by participants in Nadarzynski et al [30], who described chatbots effectively directing users either to immediate care or reassurance. They were also viewed as expansions of social networks, offering psychosocial support for isolated populations. Barnett et al [16] emphasized that chatbots might attract users needing extreme privacy and confidentiality, potentially engaging more individuals in treatment. By transparently integrating artificial intelligence (AI)–driven personalization with behavior-change theories, these digital tools not only strengthened emotional connections but also addressed key adoption barriers, creating a unified and trustworthy health-education experience.

**Figure 2.**
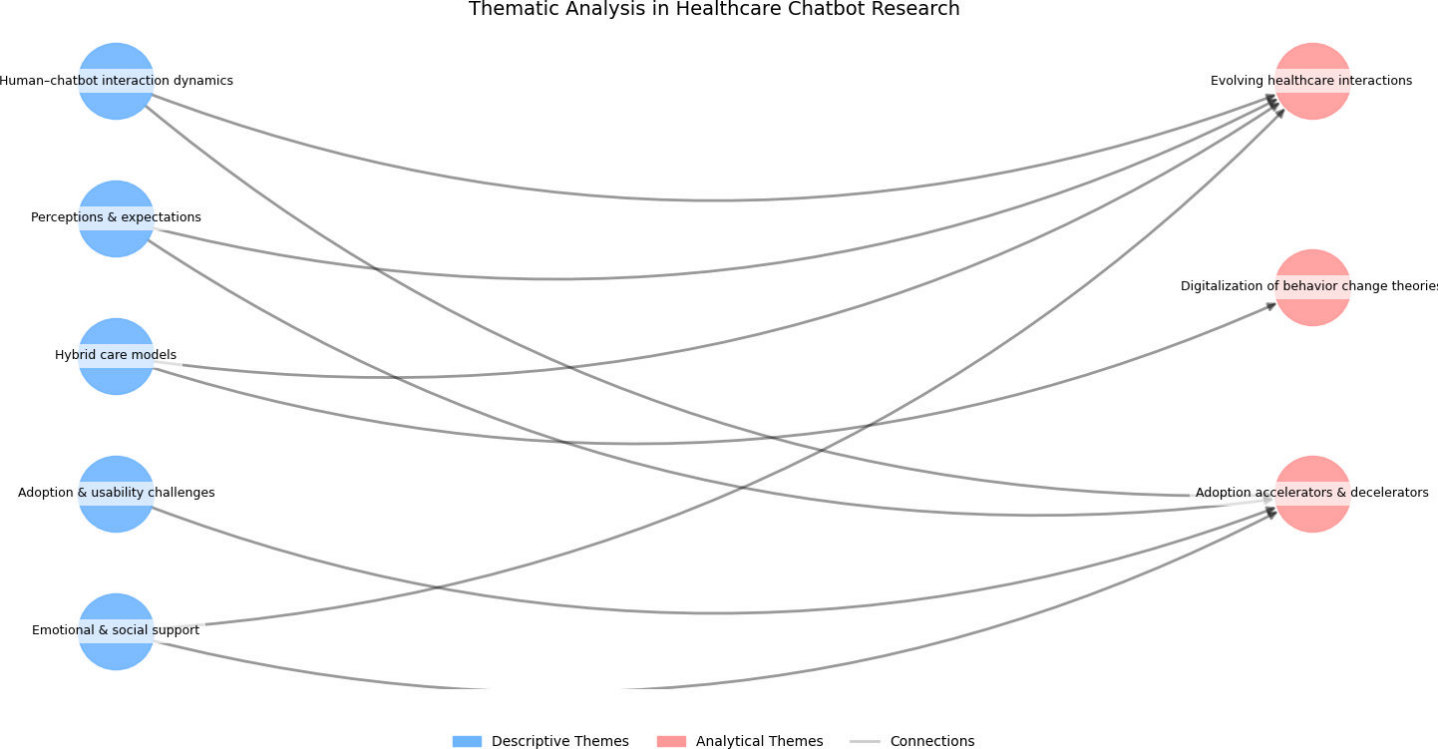
Thematic interconnections in health care chatbot research*.*

Figure 2 provides a visual representation of the descriptive and analytical themes identified in our review, illustrating how user experiences (eg, empathy, trust) intersect with theoretical frameworks and adoption factors.

### Methodological Quality and Risk of Bias

**Figure 3.**
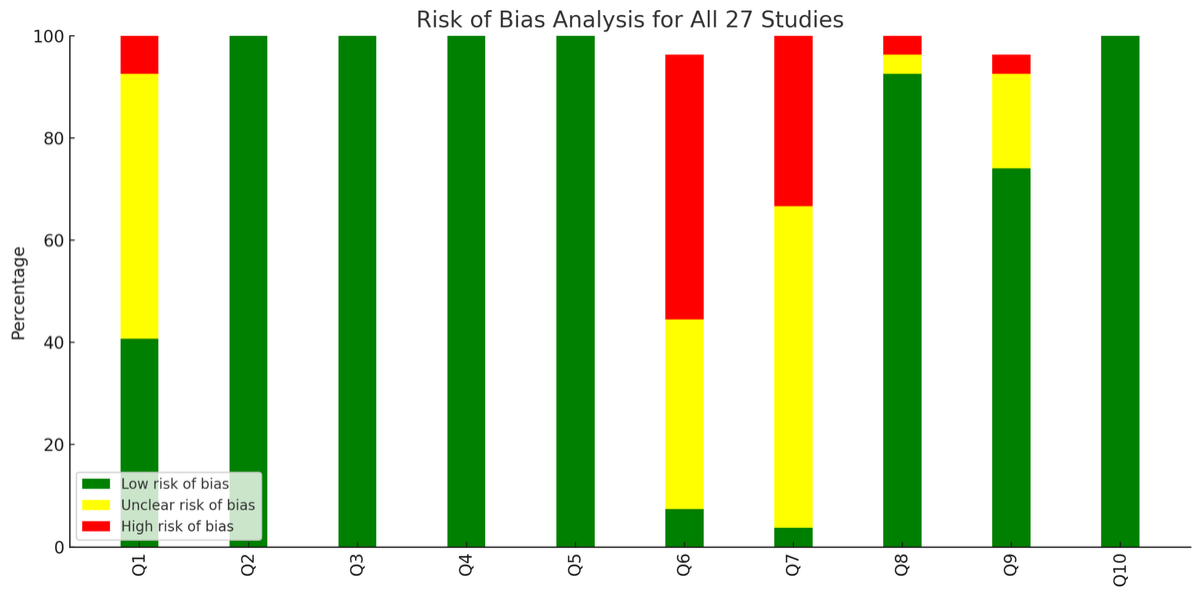
Summary of the risk of bias assessment for the 27 included studies.

The methodological rigor of the 27 studies varied. Many aligned studies aim with qualitative methods, but some lack transparency in reporting researcher biases or ethical considerations. Using the JBI Critical Appraisal Checklist, 13 studies were at low risk of bias, 12 were at unclear risk, and 2 were at high risk, primarily due to limited ethical reporting or reflexivity. Figure 3 provides a summary of the risk of bias assessment for the included studies.

## Discussion

### Principal Findings

Our synthesis reveals robust enthusiasm for chatbots as tools for health education and behavior change. In addressing RQ1 (User and Provider Experiences), we found that users value empathy and emotional support, yet they express concerns about trust and privacy. For RQ2 (Facilitators and Barriers to Adoption), 24/7 accessibility, ease of use, and personalization emerged as key facilitators, whereas trust deficits, technical glitches, and cultural misalignment were identified as major barriers. With respect to RQ3 (Role of Theoretical Frameworks), the underutilization of models such as the HBM or TAM in chatbot design has emerged as a critical gap, limiting the potential for enhanced motivational and behavioral outcomes.

Earlier meta-analyses, such as those by Laranjo et al [2], reported that chatbots can improve health outcomes, but our qualitative review complements this by emphasizing user-centered dimensions such as trust, empathy, and cultural fit—factors often overlooked in quantitative studies. For example, Nadarzynski et al [30] identified privacy as a barrier to adoption, a concern supported by our findings and detailed in the results section. Our review also identified specific barriers, such as technical errors [19], privacy gaps [16,30], and cultural mismatches [7], which previous studies have not extensively addressed. This qualitative depth highlights that chatbot success depends not only on technical functionality but also on resonating with users personally and culturally—an insight crucial for developers and policymakers aiming to foster trust and adoption across diverse populations.

Our findings offer guidance for developers to embed behavior change theories and culturally adaptive content into chatbot design. Developers should prioritize incorporating prompts that address perceived barriers (eg, tailored reminders to overcome reluctance) and foster personalized feedback loops on the basis of theoretical constructs such as the HBM or information-motivation-behavioral skills model [22]. Developers should also embed culturally relevant content and empathetic responses to build trust and engagement. This systematic integration of theories is essential for optimizing chatbot effectiveness, addressing a gap in current designs.

Healthcare providers can integrate chatbots as triage tools to complement face-to-face care and reduce routine workloads. Providers should advocate for chatbots that support, rather than replace, human interaction. Training healthcare staff in how to use chatbot systems effectively can improve both patient outcomes and provider acceptance. Our qualitative insights suggest that providers are more likely to support chatbot integration when they perceive these tools as enhancing, not threatening, their professional roles.

Policymakers should promote transparent privacy standards and fund theory-based chatbot initiatives. Regulating data security and ensuring transparent privacy policies are essential to fostering user trust. Policymakers should support the integration of theoretical frameworks into digital health tools to ensure that they are evidence-based and effective. Funding initiatives promoting culturally sensitive chatbot designs could further support adoption. The emphasis on cultural relevance and ethical considerations in our findings points to a broader need for policies that prioritize user trust and inclusivity in digital health innovations.

### Strengths and Limitations

A primary strength is the focus on qualitative evidence, offering granular insights into the emotional, cultural, and trust-based dimensions of chatbot use. The broad timescale (2018‐2023) captures recent innovations, including AI-based chatbots. However, the 2018‐2023 window may exclude older studies or those in languages beyond English/German, limiting the diversity of perspectives. Additionally, the rapid evolution of AI-driven chatbots, particularly large language models (LLMs), means that our results may not fully capture their latest affordances. The inclusion of broader conversational technologies [e.g., Biro et al [18], ; Chang et al [20], ; Svendsen et al [35], ] to offer holistic insights may reduce the specificity of findings for strictly defined chatbots. Selection bias may have influenced the findings, as many studies recruited tech-savvy participants, potentially overrepresenting positive experiences. The heterogeneity of study designs and limited focus on specific populations (eg, older adults) also constrain generalizability.

### Future Directions

Future research should examine how user perceptions (trust, empathy) evolve over time (RQ1), assess cultural influences on adoption barriers/facilitators (RQ2), and pilot systematic integration of theories into chatbot workflows (RQ3). For example, longitudinal studies should explore adherence and user engagement across time, whereas cross-cultural research could illuminate how regional dialects and health beliefs affect adoption. Methodologically, future work should strengthen transparency through preregistration and the use of qualitative reporting frameworks (eg, Consolidated Criteria for Reporting Qualitative Research), enhance ethical safeguards, adopt inclusive recruitment strategies, ensure human support during chatbot interactions, and establish clear data usage and privacy protocols. Collaborative efforts among developers, clinicians, behavioral scientists, and ethicists are essential for refining chatbot designs, ensuring data security, and embedding effective behavior

## Conclusion

This systematic review highlights robust interest in chatbots for health education and behavior change while revealing significant challenges related to privacy, cultural alignment, and theoretical underpinnings. By addressing RQ1, RQ2, and RQ3, we identified key facilitators of and barriers to chatbot adoption and underscored the importance of embedding behavior change theories into chatbot design. Although chatbots extend the reach of healthcare by providing empathetic, accessible support, their full potential remains constrained without systematic integration of behavior change models and rigorous data safeguards. Developers should prioritize user trust, cultural relevance, and theoretical grounding to enhance chatbot adoption and effectiveness. Addressing these challenges through interprofessional collaboration, ethical oversight, and ongoing research will be crucial for realizing chatbots’ transformative potential in global health systems. This review’s qualitative insights provide a foundation for designing innovative, user-centered chatbots that address diverse populations’ complex needs in digital health.

## Supplementary material

10.2196/60205 Multimedia Appendix 1 Search Strategy.

10.2196/60205 Multimedia Appendix 2 Comprehensive overview of selected studies.

10.2196/60205 Multimedia Appendix 3 Risk of bias assessment.

10.2196/60205 Checklist 1 PRISMA (Preferred Reporting Items for Systematic Reviews and Meta-Analyses) 2020 checklist.

## Abbreviations

AI: artificial intelligence
HBM: health belief model
IDEAS: Integrate, Design, Assess, and Share
JBI: Joanna Briggs Institute
PRISMA: Preferred Reporting Items for Systematic Reviews and Meta-Analyses
RQ: research question
TAM: technology acceptance model
