# Supplementary material for: User and Provider Experiences With Health Education Chatbots: Qualitative Systematic Review
Source: JMIR Hum Factors. 2025 Jun 13;12:e60205. doi: 10.2196/60205 (PMC12180679; doi:10.2196/60205)
Supplement: Multimedia Appendix 1 [file humanfactors-v12-e60205-s001.docx]

**Appendix Multimedia 1**

Database(s) search time: October 2023

Narrowing searches by time period: 2018-2023

Table: Search Strategy & Results

| **Database** | **Search Formula** | **Articles Found** | **Articles Selected** | **Notes** |
| --- | --- | --- | --- | --- |
| **PubMed** | ("chatbot" OR "conversational agent" OR "virtual assistant" OR "AI" OR "artificial intelligence") AND ("patient*" OR "health consumer*" OR "individual*") AND ("health education" OR "behavior change" OR "self-management" OR "health promotion") AND (“Qualitative Research” [Mesh] OR qualitative [tw] OR narrative [tw] OR “focus group*” [tw] OR observation* [tw] OR interview* [tw] OR “mixed method*” [tw] OR “action research” [tw] OR “content analysis” [tw] OR hermeneutic [tw] OR questionnaire [tw] OR “grounded theory” [tw] OR phenomenolog* [tw] OR explor* [tw] OR “group discussion” [tw] OR ethnographic* [tw] OR “praxis research” [tw] OR “formative evaluation” [tw] OR “process evaluation” [tw] OR participatory [tw] OR “real-world” [tw] OR “life world” [tw]) | 609 | 54 |  |
| **Cochrane** | ("chatbot" OR "conversational agent" OR "virtual assistant" OR "AI" OR "artificial intelligence") AND ("patient*" OR "health consumer*" OR "individual*") AND ("health education" OR "behavior change" OR "self-management" OR "health promotion") AND ("Qualitative Research" OR qualitative OR narrative OR "focus group*" OR observation* OR interview* OR "mixed method*" OR "action research" OR "content analysis" OR hermeneutic OR questionnaire OR "grounded theory" OR phenomenolog* OR explor* OR "group discussion" OR ethnographic* OR "praxis research" OR "formative evaluation" OR "process evaluation" OR participatory OR "real-world" OR "life world") | 81 | 8 | Simplified due to database limitations. |
| **Science Direct** | 1. ("chatbot" OR "virtual assistant") AND ("patient" OR "health consumer") AND ("health education" OR "self-management") AND ("qualitative" OR "interview" OR "focus group") <br> 2. ("chatbot" OR "virtual assistant") AND ("patient" OR "health consumer") AND ("education") AND ("qualitative research" OR "interviews" OR "focus groups" OR "participant observation") <br> 3. ("conversational agent" OR "artificial intelligence") AND ("individual") AND ("behavior change" OR "health promotion") AND ("narrative" OR "mixed method" OR "action research") | 1064 (Combined) | 86 (Combined) | Multiple, adjusted due to limitations.  Limited Boolean connectors and no wildcards supported |

Overall Results

- Total Selected on Rayyan: 148.
- Starting duplication detection: 9 duplicates found in Rayyan platform.
- 139 unique entries in Rayyan platform.

This table outlines the search strategy and initial results of the systematic review process, as detailed in the protocol, aimed at synthesizing qualitative evidence on experiences and perceptions related to chatbots and other AI tools used for health education and behavior change.

Detailed Justification for Exclusion at Full-Text Stage

| Article ID | Article Title | Reason for Exclusion | Detailed Justification |
| --- | --- | --- | --- |
| 001 | The Impact of Chatbots on Mental Health | Study design not fitting | The study was experimental rather than observational, not aligning with inclusion criteria. |
| 002 | Chatbots in Chronic Disease Management | Population not relevant | Focused on pediatric patients, whereas our review targeted adult populations. |
| 003 | Using AI for Patient Education | Outcomes not related | Main outcomes were related to AI usability, not health education or behavior change. |
| 004 | Chatbots and User Satisfaction | Irrelevant to research questions | The primary focus was on commercial metrics of user satisfaction, not health outcomes. |
| 005 | Long-term Effects of AI in Healthcare | Beyond scope of review | The study discussed long-term predictions without current data or qualitative analysis. |

Discussion of Exclusion Reasons

This rigorous exclusion process helped in minimizing bias and enhancing the reliability of the review findings. Adhering closely to the PRISMA protocol through this approach has ensured that only the most pertinent and methodologically sound studies were included, enhancing the overall quality and impact of our systematic review.
