# Supplementary material for: User and Provider Experiences With Health Education Chatbots: Qualitative Systematic Review
Source: JMIR Hum Factors. 2025 Jun 13;12:e60205. doi: 10.2196/60205 (PMC12180679; doi:10.2196/60205)
Supplement: Multimedia Appendix 2 [file humanfactors-v12-e60205-s002.pdf]

## Multimedia Appendix 2

Comprehensive Overview of Selected Studies on Chatbot Experiences and Perceptions in Health Education and Behavior Change

| Author(s) | Title | Journal | Year | DOI | Country | Aim of Study | Participants | Results |
|---|---|---|---|---|---|---|---|---|
| Baptista et al. | Acceptability of an Embodied Conversational Agent for Type 2 Diabetes Self-Management | JMIR Mhealth Uhealth | 2020 | 10.2196/17038 | Portugal | Assess the acceptability of an ECA for elderly healthcare support. | n=93, avg age 55, 44 female, 49 male | Positive feedback on usability and quality of health information. |
| Barnett et al. | Enacting 'More-Than-Human' Care: Views on Chatbots in Counseling | International Journal of Drug Policy | 2021 | 10.1016/j.drugpo.2020.102910 | Australia | Explore clients' and counselors' experiences with chatbots in counseling services. | n=28, clients and counselors with varied professional backgrounds | Recognition of chatbots' potential for providing accessible support. |
| Beaudry et al. | Getting ready for adult healthcare: Designing a chatbot to coach adolescents with special health needs through the transitions of care | Journal of Pediatric Nursing | 2019 | 10.1016/j.pedn.2019.09.004 | Canada | Design a chatbot to facilitate the transition of youth into adult healthcare. | n=13 adolescents aged between 14 and 17, seen in pediatric specialty clinics | High engagement rates and positive feedback on the chatbot's ability to provide relevant information and support. |
| Biro J, Linder C, Neyens D | The Effects of a Health Care Chatbot's Complexity and Persona on User Trust, Perceived Usability, and Effectiveness: Mixed Methods Study | JMIR Human Factors | 2023 | 10.2196/41017 | Hungary | Examine the effects of a healthcare chatbot's complexity on user satisfaction and perceived usefulness. | n=71 Initially, 74 students took part, 43 female, 28 male | Users appreciated the efficiency of chatbots, but overly complex interactions hindered satisfaction and perceived usefulness. |
| Boggiss et al. | Improving the Well-being of Adolescents With Type 1 Diabetes During the COVID-19 Pandemic: Qualitative Study Exploring Acceptability and Clinical Usability of a Self-compassion Chatbot | JMIR Diabetes | 2023 | 10.2196/40641 | Māori, New Zealand | Assess the effectiveness and acceptance of a self-compassion chatbot designed for adolescents with Type 1 Diabetes (T1D). | n=19, adolescents (aged 12-16 years) with T1D and 11 diabetes healthcare professionals, 11 female and 8 males | Adolescents rated the COMPASS chatbot positively, suggesting it for friends with T1D. Health care professionals also rated the app positively, emphasizing its clinical utility. |
| Chang IC, Shih YS, Kuo KM | Why would you use medical chatbots? Interview and survey | International Journal of Medical Informatics | 2022 | 10.1016/j.ijmedinf.2022.104827 | Taiwan | Explore the reasons for using medical chatbots from a user perspective. | 20 participants were female (70%), aged below 50, with a college/university degree(90%), and 1 – 2 years' experience of using medical chatbots | Strong correlation between the perceived usefulness of chatbots and the intention to use them for health inquiries. |
| Chen et al. | Developing a Heart Transplantation Self- | JMIR Mhealth Uhealth | 2020 | 10.2196/18999 | China | Develop a self-management chatbot for heart | n=21 subjects recruited, including 17 patients with | High satisfaction with the chatbot, noted for its usefulness in |

| | | | | | | | | |
|---|---|---|---|---|---|---|---|---|
| | Management Support Mobile Health App in Taiwan: Qualitative Study | | | | | transplantation patients. | HTx and 4 health professionals, mostly male, 51 to 60 years old, with a college education | providing personalized health management advice and support. |
| da Silva et al. | Experiences of a motivational interview delivered by a robot: Qualitative study | Journal of Medical Internet Research | 2018 | 10.2196/jmir.7737 | Australia | Examine experiences with a motivational interviewing chatbot for health behavior change. | n=20 participants from the School of Psychology's pool of research volunteers, 17 female, 3 males | Participants had positive experiences, noting the chatbot's role in supporting motivation and behavior |
| Griffin et al. | A chatbot for hypertension self-management support: User-centered design, development, and usability testing | JAMIA Open | 2023 | 10.1093/jamiaopen/ooad073 | USA | Understand information needs and perceptions toward using a chatbot for hypertension medication self-management | n=15, (8 female, 7 males) adults diagnosed with hypertension | Participants appreciated the chatbot's help in medication management, noted for its usability and interactive design. |
| Griffin et al. | Information needs and perceptions of chatbots for hypertension medication self-management: A mixed methods study | JAMIA Open | 2021 | 10.1093/jamiaopen/ooab021 | USA | Evaluate user engagement and effectiveness of a hypertension management chatbot | n=10, 5 female and 5 males; Adults with hypertension prescribed medication | Found chatbots effective in providing medication management support and improving patient engagement. |
| Han et al. | Preliminary Evaluation of a Conversational Agent to Support Self-management of Individuals Living With Posttraumatic Stress Disorder: Interview Study With Clinical Experts | JMIR Formative Research | 2023 | 10.2196/45894 | South Korea | Evaluate a conversational agent for mental health support among university students | n=10, 8 female and 2 males, clinical experts with experience in PTSD care | General agreement on the agent's potential to offer timely and accessible mental health support. |
| Hurmuz et al. | User experience and potential health effects of a conversational agent-based electronic health intervention for older adults | Internet Interventions | 2022 | 10.1016/j.invent.2022.100501 | Netherlands | Evaluate a virtual coaching system for healthy living | n=51 older adults, 70.6% female, average age of 65 years | Positive feedback on personalized advice and motivation for healthy living provided by the chatbot. |
| Kornfield et al.. | A text messaging intervention to support the mental health of young adults: User engagement and feedback from a field trial of an intervention prototype | Internet Interventions | 2023 | 10.1016/j.invent.2023.100667 | USA | Support mental health through a text messaging intervention | n=48, 66.7% female, participants engaged with mental health support texts | Positive outcomes in terms of engagement and perceived support among participants. |
| Lin FS, Shi HC, Fang KT | Exploring Pictorial Health Education Tools for Long-Term Home Care: A Qualitative Perspective | Healthcare (Basel) | 2020 | 10.3390/healthcare8030205 | Taiwan | Explore health education tools for older adults using chatbots | n=48, majority Asian, average age 21.9 years | Highlighted the potential of using pictorial aids in chatbots to enhance health education among older adults. |
| Ly KH, Ly | A fully | Internet | 2017 | 10.1016/j.in | Sweden | Systematically | n=9, mix of | Improved well- |

| | | | | | | | | |
|---|---|---|---|---|---|---|---|---|
| AM, Andersson G | automated conversational agent for promoting mental well-being: A pilot RCT using mixed methods | Interventions | | vent.2017.10.002 | | map and synthesize qualitative evidence exploring the experiences and perceptions concerning the use of chatbots in health education | women and men, average age 28.8 years | being and reduced stress among participants engaging with the chatbot. |
| Mash et al. | Evaluating the Implementation of the GREAT4Diabetes WhatsApp Chatbot to Educate People With Type 2 Diabetes During the COVID-19 Pandemic: Convergent Mixed Methods Study | JMIR Diabetes | 2022 | 10.2196/37882 | South Africa | Assess the implementation of a diabetes education chatbot during the pandemic | n=8,158 connected with the chatbot, majority finding the messages useful | High user engagement and changes in self-management behaviors noted. |
| Nadarzynski et al. | Acceptability of artificial intelligence (AI)-led chatbot services in healthcare: A mixed-methods study | Digit Health | 2019 | 10.1177/2055207619871808 | UK | Assess how acceptable AI-driven health chatbots are to users and guide the development of user-friendly health chatbots | Qualitative sub-study: n=29, Quantitative sub-study: n=215 | Identified themes affecting chatbot use and moderate overall acceptability with correlations to perceived utility and IT skills. |
| Papadopoulos et al. | Socially assistive robots in health and social care: Acceptance and cultural factors | Japan Journal of Nursing Science | 2022 | 10.1111/jjns.12523 | UK | Explore views on socially assistive robots (SARs) among nurses and midwives internationally | n=1341 from 19 countries, 85% female | Overwhelmingly positive responses about SARs' benefits, with some concerns about depersonalization and patient care. |
| Roman et al. | "Hey assistant, how can I become a donor?" The case of a conversational agent designed to engage people in blood donation | Journal of Biomedical Informatics | 2020 | 10.1016/j.jbi.2020.103461 | Brazil | Develop and assess a conversational agent to engage people in blood donation | n=50 (16 men, 34 women) | Very positive user experience, with attractiveness and stimulation scales scoring higher for female participants. |
| Schmidlen et al. | Patient assessment of chatbots for the scalable delivery of genetic counseling | Journal of Genetic Counseling | 2019 | 10.1002/jgc4.1169 | USA | Gather feedback on new communication tools, specifically chatbots, for facilitating genetic counseling | n=62 in focus groups, 20 male and 42 female participants | Participants found the chatbot’s consent process easy to understand and navigate, feedback on chatbot's name and avatar was mixed. |
| Scholten et al. | An empirical study of a pedagogical agent as an adjunct to an eHealth self-management intervention | Frontiers in Psychology | 2019 | 10.3389/fpsyg.2019.01063 | Netherlands | Investigate the support level of technology, specifically a pedagogical agent in eHealth self-management | n=230 psychology students, 16 nationalities, mainly German (70.1%) and Dutch (20.4%) | Found partial effects on feedback and autonomy, indicating varied task-related support from the GUI. |
| Siglen et al. | Ask Rosa - The making of a digital genetic | Patient Education and Counseling | 2022 | 10.1016/j.pec.2021.09.027 | Norway | Design and develop a digital | n=58 including patient representatives, | Development phase highlighted |

| | | | | | | | | |
|---|---|---|---|---|---|---|---|---|
| | conversation tool, a chatbot, about hereditary breast and ovarian cancer | | | | | conversation tool for information about hereditary breast and ovarian cancer | genetic counselors, clinical geneticists | challenges in AI-based matching of user questions with predefined answers, leading to high fallback responses. |
| Svendsen et al. | One size does not fit all: Participants' experiences of the selfBACK app to support self-management of low back pain—a qualitative interview study | BMC Musculoskeletal Disorders | 2022 | 10.1186/s12998-022-00452-2 | Denmark & Norway | Investigate the experiences of patients using the selfBACK app for self-management of low back pain | n=26, 11 female and 15 male, ages 21 to 78 | Factors influencing implementation included preferences for self-management, app tailoring, and perceived benefits; negative factors were suboptimal personalization and functionality issues. |
| Swendeman et al. | Feasibility and acceptability of mobile phone self-monitoring and automated feedback to enhance telephone coaching for people with risky substance use: The QUIT-Mobile pilot study | Journal of Addiction Medicine | 2021 | 10.1097/ADM.0000000000000707 | USA | Evaluate the feasibility, acceptability, and perceived benefits of a mobile-phone delivered intervention to prevent progression from risky drug use to addiction | n=20 primarily Black/African American and Latino men in Los Angeles | High levels of participant engagement and satisfaction; intervention deemed workable and acceptable with privacy and ease of survey completion noted. |
| ter Stal et al. | An embodied conversational agent in an eHealth self-management intervention for chronic obstructive pulmonary disease and chronic heart failure: Exploratory study in a real-life setting | JMIR Human Factors | 2021 | 10.2196/24110 | Netherlands | Investigate how an Embodied Conversational Agent's design is perceived by users in a daily life setting for chronic conditions | n=11, 7 male and 4 female, ages 49 to 83, mostly with high school or vocational education | Complexity in designing an ECA for long-term daily use highlighted; dissatisfaction with the ECA's small talk and technical issues encountered. |
| Viera et al. | A chatbot-delivered intervention for optimizing social media use and reducing perceived isolation among rural-living LGBTQ+ youth: Development, acceptability, usability, satisfaction, and utility | Internet Interventions | 2023 | 10.1016/j.invent.2023.100668 | USA | Optimize social media use and reduce isolation among rural-living LGBTQ+ youth through a chatbot-delivered intervention. | Not detailed | Initial findings suggest the chatbot was well-received with potential to significantly impact social media habits and perceptions of isolation. |
| Wang et al. | Revealing the complexity of users' intention to adopt healthcare chatbots: A mixed-method | Information Processing & Management | 2023 | 10.1016/j.ipm.2023.103444 | China | Explore the complexity of user intentions towards adopting healthcare chatbots and | n=347, mixed gender, qualitative phase involved n=12 in semi-structured interviews | Positive attitude towards healthcare chatbots noted for convenience and personalized information; |

| | | | | | | | | |
|---|---|---|---|---|---|---|---|---|
| | analysis of antecedent condition configurations | | | | | the factors influencing these decisions | | various user configurations highlighted different reasons for chatbot adoption. |
