# Supplementary material for: User and Provider Experiences With Health Education Chatbots: Qualitative Systematic Review
Source: JMIR Hum Factors. 2025 Jun 13;12:e60205. doi: 10.2196/60205 (PMC12180679; doi:10.2196/60205)

Multimedia Appendix 3

Risk of Bias Assessment

A detailed summary of the risk of bias assessment is provided in Table 1.

Table 1. Risk of Bias Assessment of Included Studies.

**Study**	**Risk of Bias**	**Comments**
Barnett et al. (2021) [9]	Low	Strong methodology and ethical considerations
Nadarzynski et al. (2019) [23]	Low	Clear methodology and participant engagement
Beaudry et al. (2019) [17]	Low	Robust design and participant representation
Chen et al. (2020) [18]	Low	Comprehensive methodology and ethical clarity
Griffin et al. (2023) [19]	Low	Strong congruence between methodology and research questions
Han et al. (2023) [20]	Low	Adequate data analysis and ethical compliance
Mash et al. (2022) [21]	Low	Thorough reporting and low risk of bias
Papadopoulos et al. (2022) [24]	Low	Strong ethical considerations and data representation
Roman et al. (2020) [25]	Low	Robust design and clear conclusions
Schmidlen et al. (2019) [26]	Low	Comprehensive analysis and ethical adherence
Scholten et al. (2019) [27]	Low	Strong methodological congruence and data interpretation
Svendsen et al. (2022) [28]	Low	High degree of congruence and participant representation
Remaining studies	Moderate	Some limitations in reporting or methodological clarity

Figure 3. Risk of bias graph: Review authors' judgments about each risk of bias item presented as percentages across all included studies.

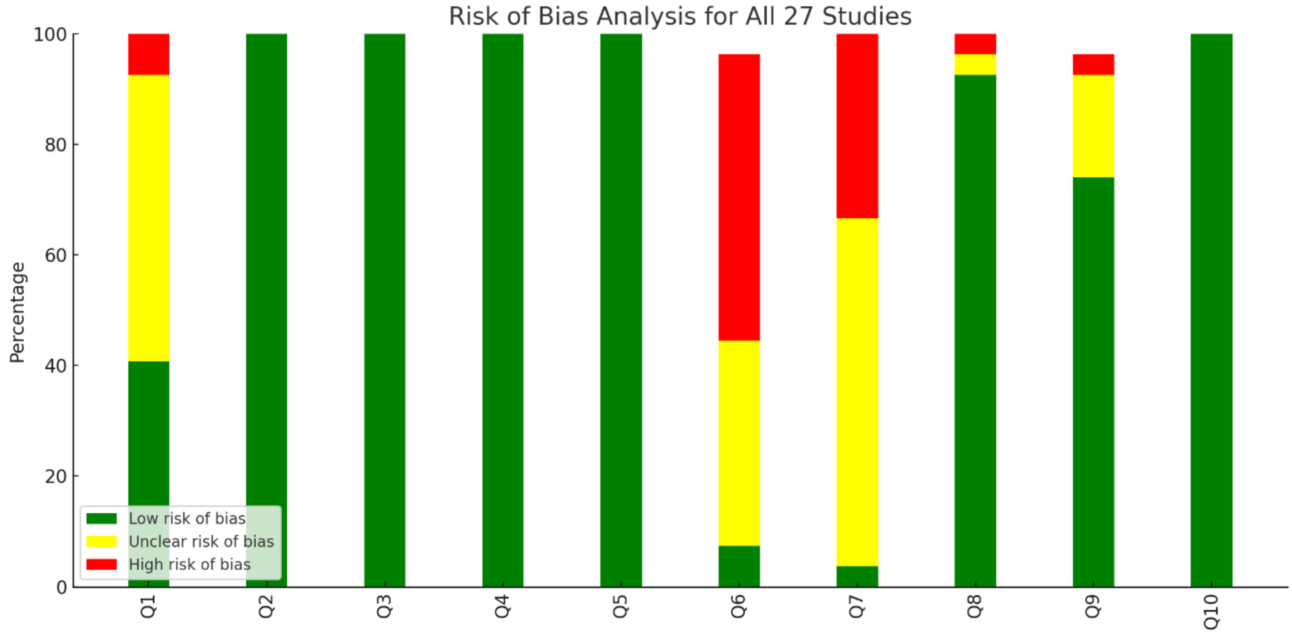

Supplement: Multimedia Appendix 3 [file humanfactors-v12-e60205-s003.pdf]
