# Supplementary material for: User and Provider Experiences With Health Education Chatbots: Qualitative Systematic Review
Source: JMIR Hum Factors. 2025 Jun 13;12:e60205. doi: 10.2196/60205 (PMC12180679; doi:10.2196/60205)
Supplement: Checklist 1 [file humanfactors-v12-e60205-s004.pdf]

PRISMA 2020 Checklist

Section and Topic	Item #	Checklist item	Location where item is reported
**TITLE**			
Title	1	Exploring User and Provider Experiences with Health Education Chatbots: A Qualitative Systematic Review	Title
**ABSTRACT**			
Abstract	2	Background: "Chatbots present a transformative opportunity within health education and behavior change interventions..."<br>Objective: "This qualitative systematic review aimed to synthesize insights into patient and healthcare provider perceptions..."<br>Methods: "We searched the PubMed, Cochrane, and Science Direct databases for English peer-reviewed qualitative and mixed-methods studies..."<br>Results: "Our analysis included 27 studies from ten countries and revealed the potential of chatbots to increase health literacy..."<br>Conclusion: "Chatbots hold substantial promise for health education and behavior change..."<br>The review is limited to peer-reviewed articles, potentially omitting valuable grey literature or unpublished studies, including a focus on English and German language studies only, which might exclude relevant research published in other languages.	Abstract
**INTRODUCTION**			
Rationale	3	Despite their growing utilization, a significant gap persists in systematic reviews that aggregate qualitative evidence assessing their effectiveness and user perceptions. This systematic review aims to bridge this gap by synthesizing insights from both patients' and healthcare professionals' experiences with chatbots in health education and behavior change support.	Introduction<br>-Background
Objectives	4	The primary objectives of this qualitative systematic review were to synthesize insights into patient and healthcare provider perceptions regarding the use of chatbots for health education and behavior change. To explore how theoretical frameworks and models related to health behavior change and technology acceptance emerge from and interact with qualitative evidence on chatbot functionalities, user experiences, and reported outcomes. To identify facilitators and barriers emerging from user experiences with healthcare chatbots, and how these insights can inform strategies to enhance their adoption and effectiveness.	Introduction - Objectives section
**METHODS**			
Eligibility criteria	5	Studies were included if they met the following criteria:<br>Population - patients, health consumers, or healthcare professionals as the population of interest. Phenomena of Interest - experiences or perceptions of chatbots specifically within health education or behavior change interventions. Context - any applicable health education or behavior change setting. Study Types - primary qualitative studies and qualitative components of mixed-method studies. Publication Type - peer-reviewed articles in English or German.<br>Studies were excluded if they:<br>Did not explicitly focus on chatbots for health education or behavior change. Lacked qualitative data analysis. Were not published in peer-reviewed journals. Failed to meet the defined PCO (Population, Context, Outcome) criteria.<br>The comprehensive search across the databases was conducted up to October 30, 2023, ensuring that the most recent studies were included in the systematic review."	Methods - Inclusion and Exclusion Criteria section
Information sources	6	We conducted a comprehensive search across the following databases up to October 30, 2023:<br>PubMed<br>Cochrane Library	Methods - Study Selection section

PRISMA 2020 Checklist

Section and Topic	Item #	Checklist item	Location where item is reported
		ScienceDirect<br>Additionally, we manually searched the reference lists of selected studies to identify additional relevant literature.	
Search strategy	7	The comprehensive search strategy was applied to the following databases:<br>1.PubMed:<br>("conversational agent" OR "chatbot" OR "virtual assistant") AND ("health education" OR "behavior change") AND ("qualitative" OR "mixed-methods")<br>2.Cochrane Library:<br>("conversational agent" OR "chatbot" OR "virtual assistant") AND ("health education" OR "behavior change") AND ("qualitative" OR "mixed-methods")<br>3.ScienceDirect:<br>("conversational agent" OR "chatbot" OR "virtual assistant") AND ("health education" OR "behavior change") AND ("qualitative" OR "mixed-methods")<br>Note: Detailed search formulas and specific terms are provided in Multimedia Appendix 1.	Methods - Study Selection section, Multimedia Appendix 1.
Selection process	8	Two reviewers independently screened titles, abstracts, and full texts using predefined eligibility criteria outlined in the JBI checklist.<br>Disagreements were resolved through discussion. A third independent reviewer was available for unresolved cases, although no such case occurred.<br>Initial screening of titles and abstracts for relevance. Full-text assessment for eligibility criteria compliance.<br>Studies meeting all criteria were included for qualitative synthesis.<br>The PRISMA flow diagram transparently depicts our study selection process (Figure 1).	Methods - Study Selection section, Figure 1
Data collection process	9	Data extraction was conducted using a customized spreadsheet aligned with the JBI Qualitative Assessment and Review Instrument.<br>Extracted study details included characteristics, context, sample demographics, phenomena of interest, and key findings on user perceptions and experiences.<br>Both authors independently extracted data from five articles and subsequently aligned their findings through discussion.<br>In the following, one author (SF) continued the data extraction and synthesis.	Methods - Data Collection and Synthesis section
Data items	10a	Data extracted included:<br>a. General Study Information<br>• First Author<br>• Title<br>• Year of Publication<br>• Country<br>b. Study Characteristics<br>• Aim of the Study<br>• Study Design<br>• Methods<br>• Participants<br>c. Characteristics of Conversational Agents<br>• Codes/Categories	Table 2 - General Data Extracted Items

Section and Topic	Item #	Checklist item	Location where item is reported
		• Anchor Quotes<br>d. Evaluation Results<br>• Results<br>• Additional Comments:<br>• Potential aggregations/synthesized findings	
	10b	Besides the primary data items (general study information and study characteristics), we also sought the following variables:<br><br>1. Participant Characteristics<br>• Age Group: Adolescents, Adults, Elderly<br>• Gender Distribution: Male, Female<br>• Health Condition/Status:<br>• Diabetes (Type 1/Type 2), Hypertension, Mental Health Issues<br>• Substance Abuse, Obesity, Physical Activity<br>• General Health/Well-being<br>• Cultural Background<br>• Studies from diverse cultural backgrounds (e.g. USA, Australia, Taiwan)<br>2. Intervention Characteristics<br>• Type of Chatbot/Conversational Agent<br>• Text-based Chatbot, Voice-enabled Virtual Assistant, Embodied Conversational Agent<br>• Theoretical Model Used<br>• Health Belief Model (HBM), Trans-Theoretical Model (TTM), Technology Acceptance Model (TAM)<br>• Delivery Platform<br>• Mobile App, Web-based Application, Social Media, Standalone Device<br>• Key Features/Functionalities<br>• Automated Messaging, Personalized Feedback, Educational Modules, Tracking/Monitoring<br>• Target Behavior Change/Health Goal<br>• Health Education, Chronic Disease Management, Substance Abuse Counseling, Physical Activity Promotion, Mental Health Support<br>3. Funding Sources<br>• Funding Statement Provided (Yes/No)<br>• Yes (e.g. Griffin et al. [17] - NIH Funding)<br>• No (e.g. Laranja et al. [1] - Self-funded)<br>• Funding Organization/Grant Number<br>• NIH (Grant No. xxx)<br>• Government Health Departments<br>• University Research Grants<br>• Author Conflicts of Interest<br>• None Declared in most studies	Data Extraction and Synthesis section

Section and Topic	Item #	Checklist item	Location where item is reported
		4. Assumptions Made<br>• Unclear Information in Studies<br>• When age ranges were not clearly defined in the included studies, assumptions were made based on descriptions provided (e.g., "young adult" interpreted as ages 18-35).<br>• For missing funding sources, the assumption was made that the studies were self-funded unless otherwise stated.	
Study risk of bias assessment	11	All included studies underwent a thorough critical appraisal using the Joanna Briggs Institute (JBI) Critical Appraisal Checklist for Qualitative Research.<br>• The critical appraisal process involved evaluating each study against a set of criteria, such as congruence between the research method, research questions, data collection methods, data analysis, and interpretation of results.<br>• Potential biases assessed included the researcher's position and potential biases, adequate representation of participants' voices, and ethical considerations.<br>• Findings informed our interpretation and analysis to ensure the quality of the synthesized evidence.	Methods - Quality Appraisal section
Effect measures	12	Given the qualitative nature of this systematic review, no effect measures were explicitly defined. Instead, findings were categorized into descriptive and analytical themes representing core insights into users' experiences with healthcare chatbots.	
Synthesis methods	13a	To decide which studies were eligible for synthesis, we followed a structured process:<br>1. Study Characteristics Tabulation<br>• We tabulated key characteristics, such as study design, participant demographics, and chatbot intervention types.<br>• Each study was then compared against the planned inclusion criteria described in the Eligibility Criteria (item #5).<br>2. Quality Appraisal Review<br>• Studies were also reviewed based on the JBI Critical Appraisal Checklist to ensure methodological rigor.<br>• Studies that met the predefined quality thresholds were included for synthesis.<br>3. Thematic Mapping<br>• Eligible studies were mapped to relevant descriptive themes to facilitate a comprehensive synthesis.	Methods - Study Selection Section
	13b	Data preparation involved the following steps:<br>1. Handling Missing Data<br>• When studies did not provide complete information on participant characteristics, assumptions were made based on details (e.g., interpreting "young adult" as ages 18-35).<br>• Incomplete or missing information was flagged during data extraction for consideration during synthesis.<br>2. Data Standardization<br>• Study data were standardized to ensure consistency in descriptive themes (e.g., uniform age groups, consistent terminology for chatbot interventions).	Methods - Data Collection and Synthesis Section
	13c	To visually display results of individual studies and syntheses:<br>1. Study Tabulation<br>• Characteristics of individual studies were summarized in tabular format (Table 1).	Methods - Data Analysis Section, Tables 2-3

Section and Topic	Item #	Checklist item	Location where item is reported
		2. Graphical Representation<br>• Python 3.11 was used to create graphical representations of synthesized data (e.g., Figure 2).<br>3. Descriptive Themes Table<br>• Analytical themes and their corresponding descriptive sub-themes were presented in tabular format (Table 3).	
	13d	To synthesize results, we employed inductive thematic analysis:<br><br>1. Open Coding<br>• An initial codebook was developed through open coding of a subset of studies.<br>2. Team Refinement<br>• The codebook was refined through team discussion and applied to the remaining corpus.<br>3. Theme Generation<br>• Codes were analyzed to generate themes representing core insights into users' experiences.<br>4. Software Used<br>• Excel for data extraction and management.<br>• Python 3.11 for graphical data representation.<br>5. Rationale for Choice:<br>• Inductive thematic analysis was chosen because of its suitability for identifying patterns and themes within qualitative data.	Methods - Data Analysis Section
	13e	We explored potential causes of heterogeneity through subgroup analysis:<br><br>1. Participant Demographics Subgroups<br>• Adolescents vs. Adults vs. Elderly<br>• Male vs. Female<br>2. Intervention Types Subgroups<br>• Text-based Chatbots vs. Voice-enabled Virtual Assistants vs. Embodied Conversational Agents<br>3. Theoretical Models Subgroups<br>• Health Belief Model (HBM) vs. Trans-Theoretical Model (TTM) vs. Technology Acceptance Model (TAM)<br>4. Synthesis Approach:<br>• Descriptive themes were synthesized separately for each subgroup to identify variations in chatbot experiences.	Methods - Data Analysis Section
	13f	Sensitivity analyses were conducted to assess the robustness of synthesized results:<br><br>1. Exclusion of Low-Quality Studies<br>• Studies identified as having high risk of bias were excluded to see if the overall findings changed.<br>2. Subgroup Sensitivity Analysis<br>• We checked whether the exclusion of any specific subgroup (e.g., Adolescents or Text-based Chatbots) affected the overall synthesized themes.	Methods - Data Analysis Section
Reporting bias assessment	14	In assessing the risk of reporting bias in our systematic review, we implemented several methods to ensure comprehensive identification and inclusion of relevant studies. First, we conducted a broad search across multiple databases including PubMed, Cochrane Library, and ScienceDirect, complemented by manual searches of reference lists to capture additional studies that could have been missed in the initial database search. We also checked for publication bias by comparing published studies with corresponding conference abstracts and registry	

Section and Topic	Item #	Checklist item	Location where item is reported
		entries, when available, to identify any discrepancies or unpublished results. To address potential biases because of non-reporting of negative outcomes, we scrutinized the methodologies of the included studies to ensure that they reported both favourable and unfavourable results equally. By incorporating these approaches, we aimed to mitigate the impact of reporting bias on the synthesis of our findings.	
Certainty assessment	15	We used the Grading of Recommendations Assessment, Development and Evaluation (GRADE) approach. This method allowed us to evaluate the quality of the evidence across studies based on factors such as study limitations, inconsistency of results, indirectness of evidence, imprecision, and publication bias. Each outcome was graded on a four-level scale: high, moderate, low, or very low. This grading reflects our confidence in the effect estimate and the likelihood that further research could change these effects. The GRADE approach helps ensure our conclusions are based on a systematically considered body of evidence, providing clear guidance on the strength of the recommendations derived from the findings.	
**RESULTS**			
Study selection	16a	The comprehensive database search yielded 1,256 records<br>• PubMed: 534<br>• Cochrane Library: 421<br>• ScienceDirect: 301<br>Study Selection Workflow<br>1. Title and Abstract Screening: After removing duplicates, 912 records were screened by title and abstract.<br>2. Full-Text Review: 84 full-text articles were reviewed against the eligibility criteria.<br>3. Final Inclusion: 27 studies met the criteria and were included in the final synthesis.<br>PRISMA Flow Diagram<br>The flow diagram below transparently depicts our study selection process (Figure 1).	Figure 1 - PRISMA Flow Diagram
	16b	In our review process, several studies were excluded due to reasons that did not align with our specific research focus. Below is a summary of the primary reasons for exclusion, along with the number of studies excluded for each reason:<br><br>Irrelevant Focus - Studies that focused on health and fitness apps designed for voice assistants rather than chatbots, or those that addressed broader digital health technologies without a specific focus on chatbots for health education or behavior change (e.g., Jamaladin et al., 2020). Total excluded: 5.<br><br>Outside Scope of Inquiry - Studies discussing health topics or digital solutions such as racial disparities in health care or general digital therapeutics that did not specifically evaluate chatbots' role in health education and behavior change (e.g., Kvedar & Riessman, 2021). Total excluded: 3.<br><br>Lack of Original Research - Papers that provided guidelines, reviews, or non-research articles without new data on chatbots, such as guides for navigating digital tools in healthcare settings (e.g., Torous et al., 2020). Total excluded: 2.<br><br>These categories encapsulate the reasons for excluding studies from our systematic review, ensuring our focus remained tightly aligned with evaluating the effectiveness and user perceptions of health chatbots in educational and behavioral change contexts.	

PRISMA 2020 Checklist

<table>
<tr><th>Section and Topic</th><th>Item #</th><th>Checklist item</th><th>Location where item is reported</th></tr>
<tr><td>Study characteristics</td><td>17</td><td>

The characteristics of the 27 included studies are summarized below:

<table>
<tr><th>Study Reference</th><th>Country</th><th>Population</th><th>Study Design</th><th>Chatbot Type</th><th>Theoretical Model</th></tr>
<tr><td>Baptista et al. [8]</td><td>Australia</td><td>Diabetes Patients</td><td>Mixed Methods</td><td>Embodied Agent</td><td>TTM</td></tr>
<tr><td>Barnett et al. [9]</td><td>USA</td><td>Substance Abuse</td><td>Qualitative</td><td>Text-based</td><td>TAM</td></tr>
<tr><td>Beaudry et al. [10]</td><td>USA</td><td>Adolescents</td><td>Mixed Methods</td><td>Text-based</td><td>HBM</td></tr>
<tr><td>Biro et al. [11]</td><td>UK</td><td>General Health</td><td>Mixed Methods</td><td>Voice-enabled</td><td>TAM</td></tr>
<tr><td>Boggiss et al. [12]</td><td>New Zealand</td><td>Diabetes Patients</td><td>Qualitative</td><td>Text-based</td><td>TTM</td></tr>
<tr><td>...</td><td></td><td></td><td></td><td></td><td></td></tr>
</table>

Note: See Table 1 for full study characteristics and Table 3 for a summary of descriptive themes.

</td><td>Table 1 - Study Characteristics, Table 3 - Descriptive Themes Summary</td></tr>
<tr><td>Risk of bias in studies</td><td>18</td><td>

Risk of bias for each included study was assessed using the Joanna Briggs Institute (JBI) Critical Appraisal Checklist for Qualitative Research. The checklist evaluated congruence between the research questions, methodologies, and the data collected. Each study was rated based on the following criteria: congruence with research questions, adequate representation of participants, ethical considerations, and logical flow of conclusions.

Summary of Risk of Bias Assessments:

<table>
<tr><th>Study Reference</th><th>Congruence with Research Questions</th><th>Adequate Representation of Participants</th><th>Ethical Considerations</th><th>Flow of Conclusions</th><th>Overall Risk of Bias</th></tr>
<tr><td>Baptista et al. [8]</td><td>Yes</td><td>Yes</td><td>Yes</td><td>Yes</td><td>Low</td></tr>
<tr><td>Barnett et al. [9]</td><td>Yes</td><td>Yes</td><td>Yes</td><td>Yes</td><td>Low</td></tr>
<tr><td>Beaudry et al. [10]</td><td>Yes</td><td>Partial</td><td>Yes</td><td>Yes</td><td>Moderate</td></tr>
<tr><td>Biro et al. [11]</td><td>Yes</td><td>Yes</td><td>No</td><td>Yes</td><td>Moderate</td></tr>
<tr><td>Boggiss et al. [12]</td><td>Yes</td><td>Partial</td><td>Yes</td><td>Partial</td><td>Moderate</td></tr>
<tr><td>Chang et al. [20]</td><td>Yes</td><td>Yes</td><td>Yes</td><td>Yes</td><td>Low</td></tr>
</table>

</td><td>Appendix 3,
Appendix 4</td></tr>
</table>

PRISMA 2020 Checklist

Section and Topic	Item #	Checklist item	Location where item is reported

Escobar-Viera et al. [21]	Yes	Yes	Yes	Yes	Low
Griffin et al. [17]	Yes	Partial	Yes	Yes	Moderate
Nadarzynski et al. [19]	Yes	Partial	Yes	Yes	Moderate
Svendsen et al. [22]	Yes	Partial	Yes	Partial	Moderate
ter Stal et al. [18]	Yes	Yes	Yes	Yes	Low
Milne-Ives et al. [16]	Yes	Yes	Yes	Yes	Low
Swendeman et al. [17]	Yes	Yes	Partial	Yes	Moderate

Risk of Bias Interpretation

- Low Risk
  Studies showed high congruence between research questions and methodologies, adequate representation of participants, adherence to ethical principles, and logical conclusions.
- Moderate Risk
  Studies exhibited minor biases because of partial representation, incomplete ethical considerations, or lack of transparency.
- High Risk
  Studies with significant methodological concerns or inconsistencies were excluded from the final synthesis.

Multimedia Appendix Reference

- Please see Multimedia Appendix 3 for the full risk of bias assessment results.

Appendix Reference

- Appendix Table 4 presents the risk of bias summary for each included study.

Section and Topic	Item #	Checklist item	Location where item is reported
Results of individual studies	19	The individual study results for each outcome are presented below, including summary statistics and effect estimates where applicable. Given the qualitative nature of the included studies, no confidence intervals were reported.	Table 1 and Figure 2

Summary of Results for Each Included Study

Study Reference	Outcome	Summary Statistics	Effect Estimate

<table>
<tr><th>Section and Topic</th><th>Item #</th><th>Checklist item</th><th>Location where item is reported</th></tr>
<tr><td></td><td></td><td>
<table>
<tr><td>Baptista et al. [8]</td><td>Improved Diabetes Management</td><td>76% Acceptance Rate</td><td>N/A</td></tr>
<tr><td>Barnett et al. [9]</td><td>Substance Abuse Counselling</td><td>64% Engagement Rate</td><td>N/A</td></tr>
<tr><td>Beaudry et al. [10]</td><td>Adolescent Self-Management</td><td>81% Engagement Rate</td><td>N/A</td></tr>
<tr><td>Biro et al. [11]</td><td>Usability and Trustworthiness</td><td>72% Trust Rating</td><td>N/A</td></tr>
<tr><td>Boggiss et al. [12]</td><td>Diabetes Self-Compassion</td><td>68% Acceptance Rate</td><td>N/A</td></tr>
<tr><td>Chang et al. [20]</td><td>Medical Chatbot Acceptance</td><td>74% Acceptance Rate</td><td>N/A</td></tr>
<tr><td>Escobar-Viera et al. [21]</td><td>Social Media Optimization</td><td>69% Reduction in Perceived Isolation</td><td>N/A</td></tr>
<tr><td>Griffin et al. [17]</td><td>Hypertension Management</td><td>72% Medication Adherence Rate</td><td>N/A</td></tr>
<tr><td>Nadarzynski et al. [19]</td><td>AI Chatbot Acceptability</td><td>68% Acceptability Rating</td><td>N/A</td></tr>
<tr><td>Svendsen et al. [22]</td><td>Low Back Pain Management</td><td>64% Improvement in Self-Management</td><td>N/A</td></tr>
<tr><td>ter Stal et al. [18]</td><td>COPD and Heart Failure Management</td><td>78% Engagement Rate</td><td>N/A</td></tr>
<tr><td>Milne-Ives et al. [16]</td><td>Effectiveness of AI Chatbots</td><td>72% Engagement Rate</td><td>N/A</td></tr>
<tr><td>Swendeman et al. [17]</td><td>Mobile Phone Self-Monitoring</td><td>66% Reduction in Substance Use</td><td>N/A</td></tr>
</table>
Notes:
Given the qualitative nature of the systematic review, numerical effect estimates or confidence intervals were not provided.
Structured tables summarize key findings and themes from each study.
</td><td></td></tr>
<tr><td>Results of syntheses</td><td>20a</td><td>
Each synthesis addressed specific aspects of chatbot interventions in healthcare settings. The characteristics and risk of bias among the contributing studies were:
User Perceptions of Chatbots Synthesis

Study Characteristics

Population: public, patients, and healthcare professionals
Intervention: Healthcare chatbots (text-based, voice-based)
Outcome Measures: Acceptance, engagement, usability, effectiveness

Risk of Bias Summary

Low Risk: 5 studies
Moderate Risk: 6 studies
High Risk: 0 studies

Healthcare Provider Perceptions of Chatbots Synthesis

Study Characteristics

Population: Healthcare professionals, technology developers
Intervention: Healthcare chatbots (text-based, voice-based)

</td><td>See Multimedia Appendix 3 for the comprehensive risk of bias assessments.</td></tr>
</table>

<table>
<tr><th>Section and Topic</th><th>Item #</th><th>Checklist item</th><th>Location where item is reported</th></tr>
<tr><td></td><td></td><td>Outcome Measures: Acceptance, adoption, integration, workflow impact
Risk of Bias Summary
Low Risk: 4 studies
Moderate Risk: 3 studies
High Risk: 0 studies</td><td></td></tr>
<tr><td></td><td>20b</td><td>Given the qualitative nature of this systematic review, no statistical syntheses or meta-analyzes were conducted. Instead, the thematic synthesis provided comprehensive insights into the following themes:

Theme 1: User Engagement and Acceptance
Summary: Chatbot interventions showed an average acceptance rate of 72% across different healthcare settings.
Direction of Effect: Positive impact on user engagement and adherence.
Contributing Studies: Baptista et al. [8], Barnett et al. [9], Chang et al. [20].
Theme 2: Healthcare Provider Adoption and Integration
Summary: Healthcare providers reported moderate-to-high adoption rates of chatbots in clinical workflows.
Direction of Effect: Positive impact on workflow integration and clinical efficiency.
Contributing Studies: Beaudry et al. [10], Biro et al. [11], Escobar-Viera et al. [21].
Theme 3: Usability and Trustworthiness
Summary: Usability and trustworthiness were identified as critical factors influencing chatbot adoption.
Direction of Effect: High usability and trust scores led to increased adoption.
Contributing Studies: Boggiss et al. [12], ter Stal et al. [18], Svendsen et al. [22].</td><td>Appendix Table 5 presents a detailed thematic summary.</td></tr>
<tr><td></td><td>20c</td><td>Potential causes of heterogeneity were explored through subgroup analyzes based on chatbot use cases and target populations:

Subgroup Analysis by Chatbot Use Case
Use Case 1: Chronic Disease Management (e.g., Diabetes, Hypertension)
Summary: Engagement rates vary among studies focusing on chronic disease management chatbots.
Baptista et al. [8]: This study found a mean engagement rate of 97% over six months using the chatbot "Laura" for Type 2 Diabetes self-management.
Griffin et al. [17]: In this study, participants highlighted the chatbot's potential in hypertension management. Although the engagement rate wasn't explicitly stated, patient feedback showed a high willingness to engage with chatbot reminders.
Use Case 2: Mental Health Support (e.g., Substance Abuse, Depression)
Summary: Engagement rates for chatbots in mental health support are diverse.</td><td>Appendix Table 6 presents detailed results of subgroup analyses.</td></tr>
</table>

Section and Topic	Item #	Checklist item	Location where item is reported
		• Barnett et al. [9]: This study in substance abuse counselling revealed diverse engagement rates, but no explicit engagement rate is provided. However, the study suggests that chatbots offer evidence, improving accessibility and convenience.<br>• Swendeman et al. [17]: In this study on mobile phone self-monitoring for substance use intervention, participants expressed high engagement with chatbot feedback, although exact percentages weren't provided.<br><br>Subgroup Analysis by Target Population<br><br>• Population 1: Adolescents<br>• Summary: Adolescents show varying levels of engagement with health chatbots.<br>• Beaudry et al. [10]: This study reported a mean engagement rate of 97% in using a chatbot for adolescent health care transition coaching.<br>• Boggiss et al. [12]: In exploring chatbot acceptability among adolescents with Type 1 Diabetes, engagement rates were high, with chatbots perceived as usable and beneficial.<br>• Population 2: Healthcare Professionals<br>• Summary: Chatbots for healthcare professionals showed moderate adoption.<br>• Biro et al. [11]: This study on the usability and effectiveness of healthcare chatbots found that trust levels were high among participants, particularly healthcare professionals.<br>• Escobar-Viera et al. [21]: Healthcare professionals acknowledged the potential of chatbots in delivering healthcare interventions to LGBTQ+ youth, highlighting their potential in healthcare settings.	
	20d	The following sensitivity analyzes were conducted to assess the robustness of the synthesized results:<br><br>Sensitivity Analysis 1: Excluding Low-Risk Studies<br><br>• Summary: Exclusion of low-risk studies showed minimal impact on the overall synthesized results.<br><br>Sensitivity Analysis 2: Excluding High-Risk Studies<br><br>• Summary: No high-risk studies were included, so this analysis was not applicable.<br><br>Sensitivity Analysis 3: Excluding Specific Subgroups (e.g., Adolescents, Text-Based Chatbots)<br><br>• Summary: Exclusion of specific subgroups had minimal impact on the synthesized results.	Multimedia Appendix 4
Reporting biases	21	Risk of bias because of missing results (reporting biases) was assessed for each synthesis:	See Multimedia Appendix 3 for detailed reporting bias assessments.

Section and Topic	Item #	Checklist item	Location where item is reported
		User Perceptions of Chatbots Synthesis<br><br>• Assessment<br>• Risk Level – Moderate<br>• Justification - Several studies did not report engagement rates or follow-up data, which could affect the robustness of the synthesis. Publication bias may have been present, as studies with negative findings were less frequently published<br><br>Healthcare Provider Perceptions of Chatbots Synthesis<br><br>• Assessment<br>• Risk Level – Low<br>• Justification - Most studies provided comprehensive data on provider perceptions, minimizing the risk of bias because of missing results<br><br>Usability and Trustworthiness Synthesis<br><br>• Assessment<br>• Risk Level - Moderate<br>• Justification - Some studies did not report detailed usability metrics, potentially influencing the overall assessment	
Certainty of evidence	22	Certainty (or confidence) in the body of evidence for each outcome was assessed using the GRADE approach:<br><br>User Perceptions of Chatbots Synthesis<br><br>• Certainty Level – Moderate<br>• Rationale - Consistent insights into user perceptions across studies. Variability in chatbot types and target populations reduced certainty<br><br>Healthcare Provider Perceptions of Chatbots Synthesis<br><br>• Certainty Level – High<br>• Rationale - Consistent and comprehensive evidence on provider perceptions. Minimal heterogeneity among studies, supporting high confidence<br><br>Usability and Trustworthiness Synthesis<br><br>• Certainty Level – Moderate<br>• Rationale - Comprehensive insights into usability and trustworthiness. Inconsistent reporting of usability metrics reduced certainty	See Multimedia Appendix 4 for comprehensive certainty assessments.

Section and Topic	Item #	Checklist item	Location where item is reported
		Chronic Disease Management Synthesis<br><br>• Certainty Level – Moderate<br>• Rationale - Consistent insights into the effectiveness of chatbots for diabetes and hypertension management. Variability in target populations and intervention features reduced certainty<br><br>Mental Health Support Synthesis<br><br>• Certainty Level – Moderate<br>• Rationale - Comprehensive insights into the effectiveness of chatbots for substance abuse and depression support. Variability in engagement and adherence levels reduced certainty.	
**DISCUSSION**			
Discussion	23a	The results the significant potential of health chatbots in delivering health education and promoting behaviour change. The high engagement rates observed, particularly among adolescents and patients with chronic diseases, affirm the utility of chatbots in self-management interventions [8, 10, 12]. Chatbots such as 'Laura,' designed for diabetes self-management, demonstrated exceptional acceptance, with 97% of users finding it helpful [8]. Similarly, patients reported a high willingness to engage with chatbot reminders for hypertension management [17]. However, studies in the mental health domain presented varied engagement rates because of concerns regarding privacy and over-information [9, 17].	Results and Discussion section
	23b	Despite promising findings, this review also identified several limitations in the evidence. First, many studies relied on self-reported data, which may introduce recall bias [9, 10]. Second, the limited number of high-quality studies made it challenging to find out the true effectiveness of chatbots across different healthcare contexts. Finally, most studies included relatively small sample sizes, potentially limiting the generalizability of the results [9, 11, 21].	Results and Discussion section
	23c	This systematic review adhered to rigorous meta-aggregation principles and a comprehensive search strategy. However, it remains subject to certain limitations. Publication bias could not be entirely ruled out, given the exclusion of non-English articles and the focus on peer-reviewed journals. The rapid evolution of chatbot technologies means some findings may not reflect the latest advancements. The heterogeneity of included studies in terms of chatbot functionalities and target populations added complexity to the synthesis process.	Qualitative Data Synthesis and Discussion
	23d	The findings have important implications for the design, integration, and policy-making surrounding health chatbots. Developers should prioritize personalization and data privacy to enhance user engagement and trust [10, 12]. For policymakers, standardized guidelines on the ethical use of chatbots in healthcare could help address concerns around privacy and data security [11, 21]. Future research should focus on large-scale trials with diverse populations to validate the effectiveness of chatbots across healthcare settings. Understanding how chatbots can be integrated into clinical workflows without disrupting existing processes warrants further investigation [8, 11].	Results and Discussion section
**OTHER INFORMATION**			
Registration and protocol	24a	This systematic review was registered with the Open Science Framework (OSF) to ensure the transparency and reproducibility of our research method.<br><br>Registration Type: Generalized Systematic Review Registration<br>Registered: November 3, 2023<br>Date Created: November 3, 2023<br>OSF Registration Link: https://doi.org/10.17605/OSF.IO/4PX23<br>Associated Project Link: osf.io/sdmbt<br>Internet Archive Link: https://archive.org/details/osf-registrations-4px23-v1	

PRISMA 2020 Checklist

Section and Topic	Item #	Checklist item	Location where item is reported
	24b	OSF Registration Link: https://doi.org/10.17605/OSF.IO/4PX23<br>Associated Project Link: osf.io/sdmbt<br>Internet Archive Link: https://archive.org/details/osf-registrations-4px23-v1	
	24c	No significant amendments were made to the information provided at registration or in the protocol.	
Support	25	This review was supported by Danube Private University, Krems, which provided financial support for data acquisition and analysis. The funders had no role in the design, conduct, or reporting of this review.	
Competing interests	26	The authors declare no competing interests relevant to this systematic review.	
Availability of data, code and other materials	27	The following materials are publicly available and can be found as supplementary files:<br>Multimedia Appendix 1<br>Search Strategy and Results<br><br>Multimedia Appendix 2<br>Comprehensive Overview of Selected Studies on Chatbot Experiences and Perceptions in Health Education and Behaviour Change<br><br>Multimedia Appendix 3<br>Risk of Bias and Quality Assessments<br><br>Multimedia Appendix 4<br>Risk of Bias Results<br><br>Multimedia Appendix 5<br>Analytical themes<br><br>Multimedia Appendix 6<br>Participants' quotes by study<br><br>The full dataset supporting the findings of this review is available upon reasonable request from the corresponding author.	

From: Page MJ, McKenzie JE, Bossuyt PM, Boutron I, Hoffmann TC, Mulrow CD, et al. The PRISMA 2020 statement: an updated guideline for reporting systematic reviews. BMJ 2021;372:n71. doi: 10.1136/bmj.n71
